# Dermatology

**Published:** 1908-02-22

**Authors:** 


### Dermatology.

### Psoriasis in Three Generations.

Among the cases at the Wednesday Skin Clinic at the Prince of Wales's General Hospital, Tottenham, the following presented several features of interest:—

A man, aged 43, and his son, aged ten, both presented typical psoriasis, the former having been first affected at the age of eighteen, while the boy was first attacked two years ago upon the elbows. His scalp and legs were also covered with the scaly eruption. The paternal grandfather and an uncle also suffered from a "similar eruption." The father was gouty, but there was no history of rheumatism in the family. Both patients appeared to have good bodily health, so that these cases would seem to bear out Hebra's dictum that psoriasis is a "disease of the healthy." It not infrequently happens, however, that psoriatics present some or other considerable deviation from the normal, and the statistics published by Abraham show that a very fair number of the cases are, or have been recently, affected by some other complaint, such as severe dyspepsia, constipation, gout or rheumatism, or else that they have been exposed to some debilitating influence. Hence, it is often necessary to give internal treatment, not of a specific character—for there is no specific remedy for psoriasis—directed towards the constitutional trouble that may coexist with the cutaneous disorder.

